# Rabphilin involvement in filtration and molecular uptake in *Drosophila* nephrocytes suggests a similar role in human podocytes

**DOI:** 10.1242/dmm.041509

**Published:** 2020-09-21

**Authors:** Estela Selma-Soriano, Beatriz Llamusi, Juan Manuel Fernández-Costa, Lauren Louise Ozimski, Rubén Artero, Josep Redón

**Affiliations:** 1 Translational Genomics Group, Incliva Health Research Institute, 46010 Valencia, Spain; 2 Interdisciplinary Research Structure for Biotechnology and Biomedicine (ERI BIOTECMED), University of Valencia, 46100 Valencia, Spain; 3 CIPF-INCLIVA Joint Unit, 46010 Valencia, Spain; 4 Hypertension Unit, Hospital Clínico Universitario, 46010 Valencia, Spain

**Keywords:** *Drosophila* nephrocyte, *Rabphilin*, Endocytic pathway, Chronic kidney disease, Slit diaphragm, Labyrinthine channels

## Abstract

*Drosophila* nephrocytes share functional, structural and molecular similarities with human podocytes. It is known that podocytes express the rabphilin 3A (*RPH3A*)*-RAB3A* complex, and its expression is altered in mouse and human proteinuric disease. Furthermore, we previously identified a polymorphism that suggested a role for RPH3A protein in the development of urinary albumin excretion. As endocytosis and vesicle trafficking are fundamental pathways for nephrocytes, the objective of this study was to assess the role of the *RPH3A* orthologue in *Drosophila*, *Rabphilin* (*Rph*), in the structure and function of nephrocytes. We confirmed that *Rph* is required for the correct function of the endocytic pathway in pericardial *Drosophila* nephrocytes. Knockdown of *Rph* reduced the expression of the *cubilin* and *stick and stones* genes, which encode proteins that are involved in protein uptake and filtration. We also found that reduced *Rph* expression resulted in a disappearance of the labyrinthine channel structure and a reduction in the number of endosomes, which ultimately leads to changes in the number and volume of nephrocytes. Finally, we demonstrated that the administration of retinoic acid to IR-Rph nephrocytes rescued some altered aspects, such as filtration and molecular uptake, as well as the maintenance of cell fate. According to our data, Rph is crucial for nephrocyte filtration and reabsorption, and it is required for the maintenance of the ultrastructure, integrity and differentiation of the nephrocyte.

## Abstract (precis)

**Summary:** Rabphilin is necessary for the maintenance of *Drosophila* nephrocytes. Knockdown of Rabphilin causes deregulation of the filtration and the reabsorption processes, which are restored by the administration of retinoic acid.

## INTRODUCTION

The rabphilin 3A (*RPH3A*) gene encodes a Rab small GTPase family effector protein that has been implicated in vesicle docking/fusion reactions and the regulation of exocytosis and endocytosis processes in the central nervous system (Burns et al., 1998). *RPH3A* and Rab3A, a small G protein family member, complex with each other and are restrictedly expressed in neurons and podocytes, in which they are found around vesicles contained in the foot-processes (Rastaldi et al., 2003). *RPH3A* expression is altered in mouse and human proteinuric disease, suggesting a role for this protein in glomerulopathies (Rastaldi et al., 2003). Moreover, several human proteinuric diseases show enhanced *RAB3A* expression (Rastaldi et al., 2003). Other data that support the importance of RPH3A in human proteinuric diseases are combined genomic and metabolomic analyses that have previously associated increased urinary albumin excretion (UAE) in the general population (Marrachelli et al., 2014; Hwang et al., 2007) with an *RPH3A* gene polymorphism. This increase was independent of known factors that can influence UAE, such as hypertension and diabetes (Marrachelli et al., 2014). Importantly, the structural and functional impact of altered *RPH3A* expression on podocytes is still unknown.

*Drosophila* provides a suitable experimental model as it combines podocyte and renal proximal tubule functions in the same cell, the nephrocyte (Fu et al., 2017a). This makes *Drosophila* nephrocytes interesting for *in vivo* studies that can be difficult to perform in mammals due to a lack of accessibility. These nephrocytes that form the *Drosophila* excretory system (Denholm and Skaer, 2009; Helmstädter and Simons, 2017) are the cells involved in the removal of waste products from the haemolymph and Malpighian tubules. The function of Malpighian tubules, regarded as analogous to the renal tubular system, is to form urine for the excretion of toxic substances (Helmstädter and Simons, 2017; Helmstädter et al., 2017). There are two distinct nephrocyte populations: the garland cell nephrocytes around the oesophagus (binucleate) and the pericardial nephrocytes (mononucleate) situated along the heart. Both types of nephrocytes are functionally, structurally and molecularly similar to human podocytes (Helmstädter and Simons, 2017; Helmstädter et al., 2017; Na and Cagan, 2013; Fu et al., 2017b; Weavers et al., 2009; Hochapfel et al., 2017; Hermle et al., 2017; Zhuang et al., 2009).

As in mammalian podocytes, the nephrocyte basement membrane carries out charge-selective filtration (Hermle et al., 2017). The second filtration barrier is the nephrocyte diaphragm (ND) formed by Kirre and Stick and stones (Sns), which are the *Drosophila* orthologues of the mammalian slit diaphragm (SD) proteins Neph1 and nephrin, respectively. The nephrocyte membrane forms invaginations called labyrinthine channels, in which most endocytic uptake occurs (Helmstädter and Simons, 2017; Zhuang et al., 2009; Tutor et al., 2014). Kirre and Sns seal these invaginations to form 40-nm-wide slit pores, which are the entry to the labyrinthine channels (Zhuang et al., 2009). Cubilin (Cubn) and Amnionless are located in the innermost part of the labyrinthine channels, in which these proteins have an important role in endocytosis (Zhang et al., 2013a,b). Another requirement for the differentiation and maintenance of nephrocytes is the expression of the transcription factor *Krüppel-like factor 15* (*Klf15*) (Ivy et al., 2015; Mallipattu et al., 2012). In *Klf15* mutants, both garland cells and pericardial nephrocytes are absent, although the *Klf15* mutant flies exhibit normal lifespan (Ivy et al., 2015).

In this study, we report the expression of *Rph*, the *Drosophila* orthologue of *RPH3A*, in the pericardial nephrocytes colocalizing with molecular markers of both endocytic and exocytic pathways. Nephrocyte-targeted interference of *Rph* expression blocked the endocytosis of different sizes of dextrans, impaired toxin removal and reduced the expression of essential nephrocyte genes, such as *Cubn* and *sns*. Importantly, *Rph* silencing disrupted the ultrastructure of the nephrocyte, abrogated the labyrinthine channels, reduced the number of endosomes and ultimately resulted in *Drosophila* nephrocyte loss. Our results indicate that *Rph* is necessary for the endocytic pathway and *Rph* loss of function has a strong impact on the structure, function and differentiation of nephrocytes. The role of Rph in the maintenance of the structure and function of nephrocytes opens a window to explore the contribution of *RPH3A* gene polymorphisms to the risk of developing chronic kidney disease.

## RESULTS

### *Rph* is expressed in pericardial nephrocytes

**Fig. 1.**
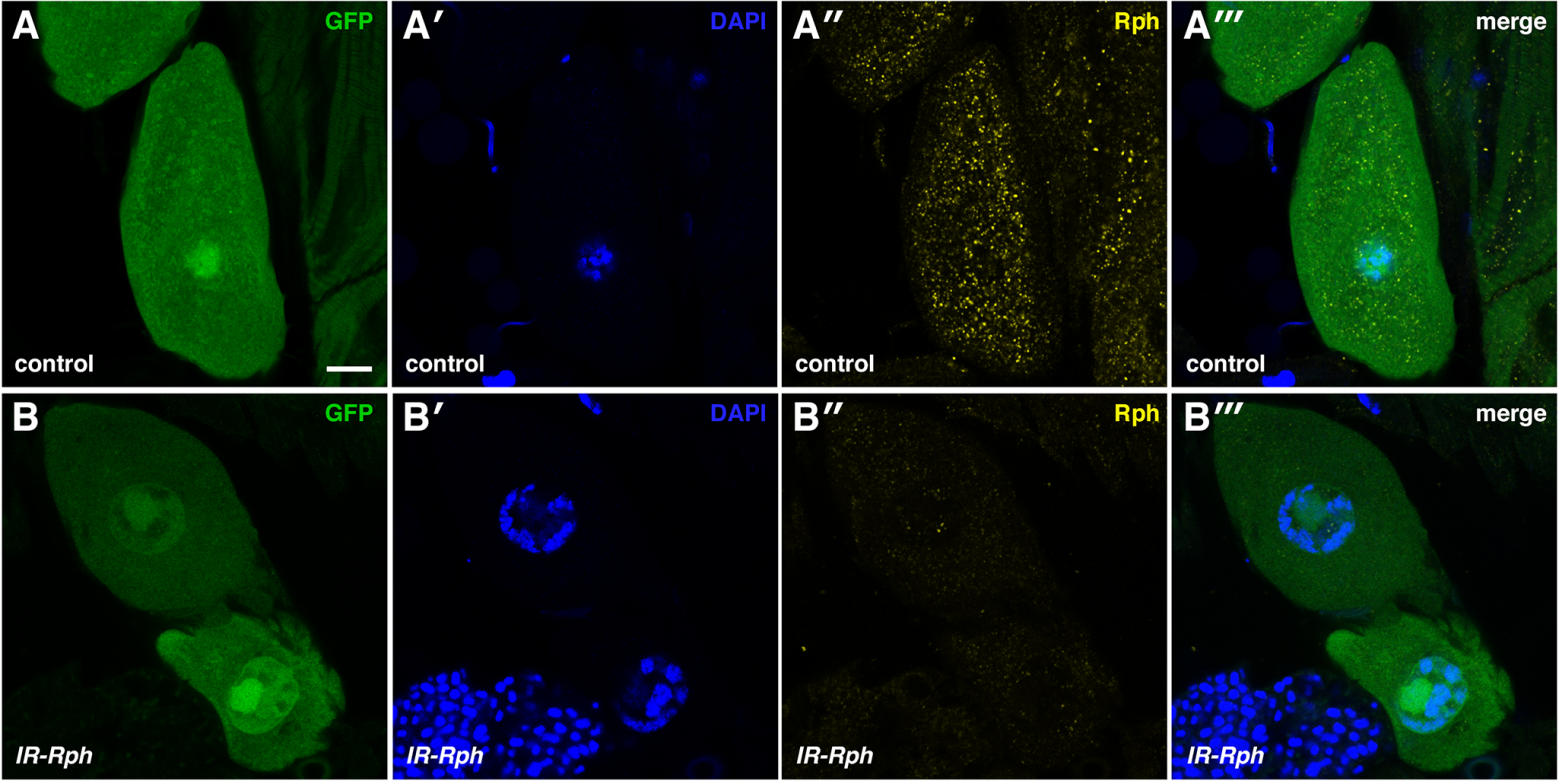
***Rph* is expressed in adult *Drosophila* pericardial nephrocytes.** (A,B) Representative confocal images of adult flies expressing the GFP reporter (green in A and B) under the control of the *Hand-Gal4* driver. Immunostaining with anti-Rph antibody (A″,A‴,B″,B‴) revealed Rph presence in the pericardial nephrocytes of control flies (A-A‴) but not in flies expressing an *Rph* RNAi construct (B-B‴). Nuclei were counterstained with DAPI (blue in A′,A‴,B′ and B‴). Scale bar: 10 μm.

In order to use *Drosophila* as a model to study the potential role of *Rph* in nephrocytes, we first investigated the expression and localization of this protein in pericardial nephrocytes of adult 7-day-old female flies. To label the nephrocytes, we used the UAS-Gal4 binary system to express the GFP marker under the control of the *Hand-Gal4* driver (Sellin et al., 2006), which promotes expression in the cardiomyoblasts and pericardial cells starting at embryonic stage 12. Semi-intact heart preparations of *Hand-Gal4>UAS-GFP* flies allowed the direct visualization of pericardial nephrocytes under a fluorescence confocal microscope. To detect Rph, we took advantage of the high degree of evolutionary conservation of this protein (Fukuda et al., 2004) and performed immunofluorescence with a commercial rabbit polyclonal anti-Rph3A antibody generated against a synthetic peptide (amino acids 1-18) corresponding to rat *Rph3A.* In control flies, the immunofluorescence showed punctate localization of the signal in pericardial nephrocytes (*Hand-Gal4>UAS-GFP*; Fig. 1A-A‴). The signal decreased when the same immunofluorescence was performed in nephrocytes expressing a *Rph* RNAi construct (Perkins et al., 2015), demonstrating the specificity of the antibody (*Hand-Gal4>UAS-GFP UAS-IR-Rph*, abbreviated *IR-Rph*; Fig. 1B-B‴, Fig. S1A). Moreover, quantification of the *Rph* transcripts by RT-qPCR from manually isolated adult *Drosophila* hearts, which are enriched in nephrocytes, confirmed a decrease of up to 50% of the normal expression of this gene in the *Rph* RNAi sample compared with the control (Fig. S1B). Following the same immunofluorescence assay, we demonstrated that Rph is also expressed in nephrocytes from control larvae, and its signal is decreased in nephrocytes from *Rph* knockdown larvae (Fig. S2). To validate the RNAi *Rph* effect, we studied *Rph* expression in different interference lines. We observed that RNAi lines from different sources exhibit different levels of Rph signal (Fig. S3A-C″,D). In the three lines analyzed, Rph signal was reduced but the strongest effect was detected with line 25950 from Bloomington *Drosophila* Stock Center (BDSC), which was used as reference *IR-Rph* in the following experiments (Fig. 1, Fig. S1A,B).

### *Rph* contributes to maintaining adult nephrocyte cell fate

**Fig. 2.**
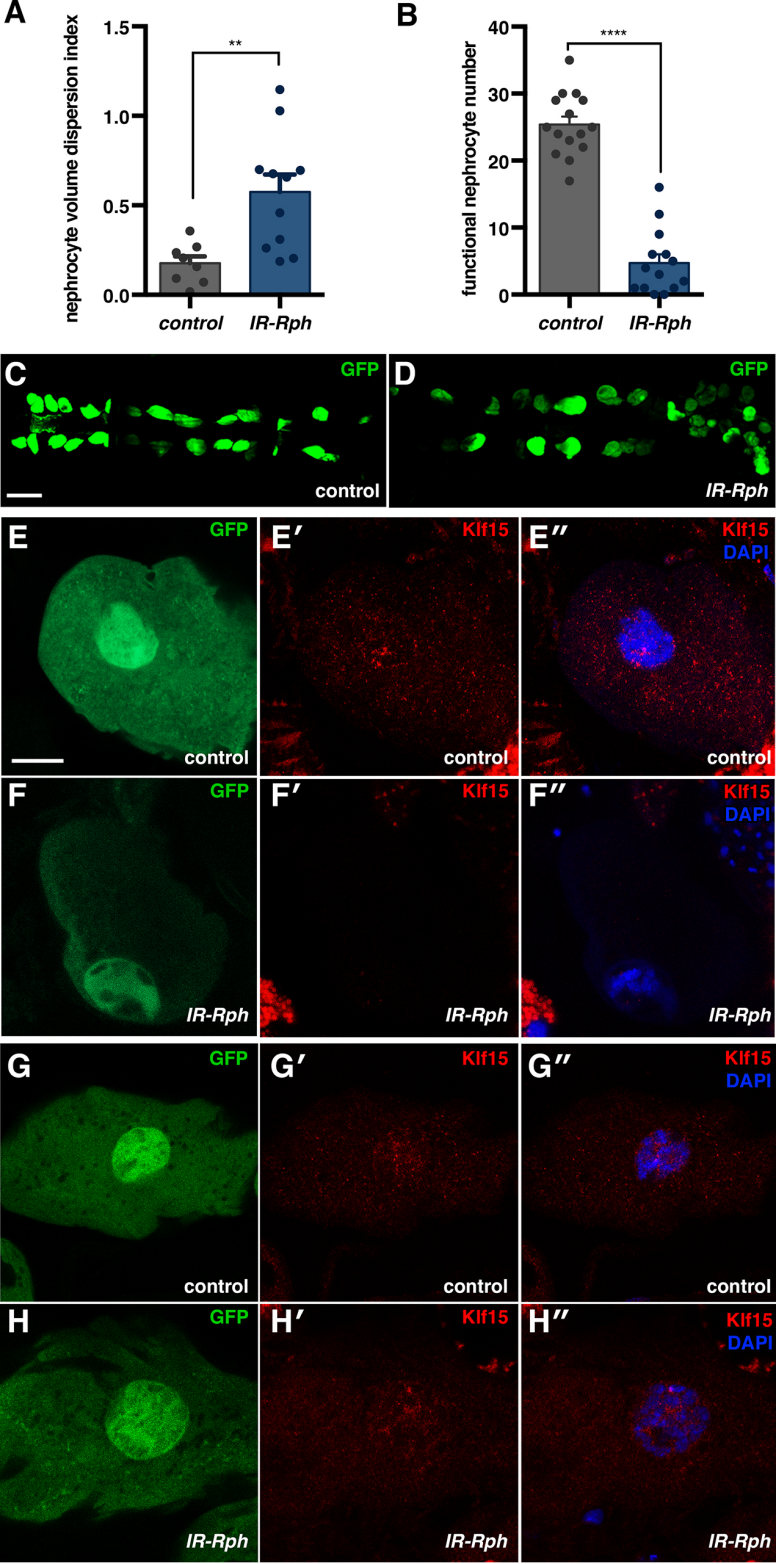
**Adult pericardial nephrocytes require *Rph* function for the maintenance of their fate.** (A,B) Quantification of the nephrocyte volume dispersion index (A) and the average number of functional pericardial nephrocytes (B). Individual data points are indicated. Data are mean±s.e.m. Two-tailed unpaired Student's *t*-test. **P<0.01, ****P<0.0001. (C-H) Fluorescence confocal microscopy images of control (*Hand-Gal4>UAS-GFP*; C,E-E″,G-G″) and *IR-Rph* pericardial nephrocytes (*Hand-Gal4>UAS-GFP IR-Rph*; D,F-F″,H-H″) of 1-week (C-F″) and 1-day-old flies (G-H″) labelled with GFP (green) and immunostained with an anti-Klf15 antibody (red). Nuclei were counterstained with DAPI (blue in E″,F″,G″,H″). Scale bars: 100 μm (C,D); 10 μm (E-H″).

To assess the role of Rph in *Drosophila* nephrocytes, we counted the number of functional pericardial nephrocytes (GFP^+^ cells with the nuclei intact) and measured cell volume in both control and *IR-Rph* flies, as these two parameters have previously been demonstrated to be informative about cell fate and activity, respectively (Ivy et al., 2015; Fu et al., 2017c). The volume dispersion index, calculated as SD volume/ median in 1-week-adult flies, was robustly increased in *IR-Rph* flies (Fig. 2A), thus reflecting an abnormal variability in the mean volume of *Rph* knockdown nephrocytes (Fig. 2C,D). Furthermore, the average number of functional nephrocytes was significantly reduced in *IR-Rph* flies compared with control flies (Fig. 2B, Fig. S3E). To assess whether the loss of nephrocytes in the *IR-Rph* flies was anticipated by defective differentiation during development, we studied the expression of the *Drosophila* orthologue of *KLF15* (*Klf15*), which is critical for the development and differentiation of *Drosophila* nephrocytes, and can be used as a terminal differentiation marker (Ivy et al., 2015; Mallipattu et al., 2012, 2017). Immunofluorescence using a polyclonal antibody against a synthetic peptide of human KLF15 (amino acids 45-94), showed *Klf15* expression in the nuclei of control nephrocytes (Fig. 2E-E″), whereas the signal was absent in most of the nephrocytes of the *IR-Rph* 1-week-old flies (Fig. 2F-F″, Fig. S4A). In contrast, *Klf15* expression was normal in *IR-Rph* nephrocytes of 1-day-old flies (Fig. 2G-H″), indicating that the expression of this transcription factor is lost with time in *IR-Rph* nephrocytes. The specificity of this KLF15 antibody was tested in control and retinoic acid (RA)-supplemented nephrocytes (Fig. S5). Consistent with the well-known upregulation of *Klf15* expression by RA, we observed that the levels of Klf15 in control flies fed with RA were robustly increased compared with the nephrocytes of control flies fed with standard food (Fig. S4B, Fig. S5).

Importantly, the GFP signal pattern in the 1-week-old nephrocytes was lighter compared with control nephrocytes at the same age (Fig. 2E,F, Fig. S3F). These results show that decreased levels of *Rph* in the pericardial nephrocytes promotes dedifferentiated nephrocytes, which are ultimately lost. Therefore, *Rph* is necessary for sustained nephrocyte cell fate.

### Reduced expression of *Rph* alters vesicular trafficking in nephrocytes

**Fig. 3.**
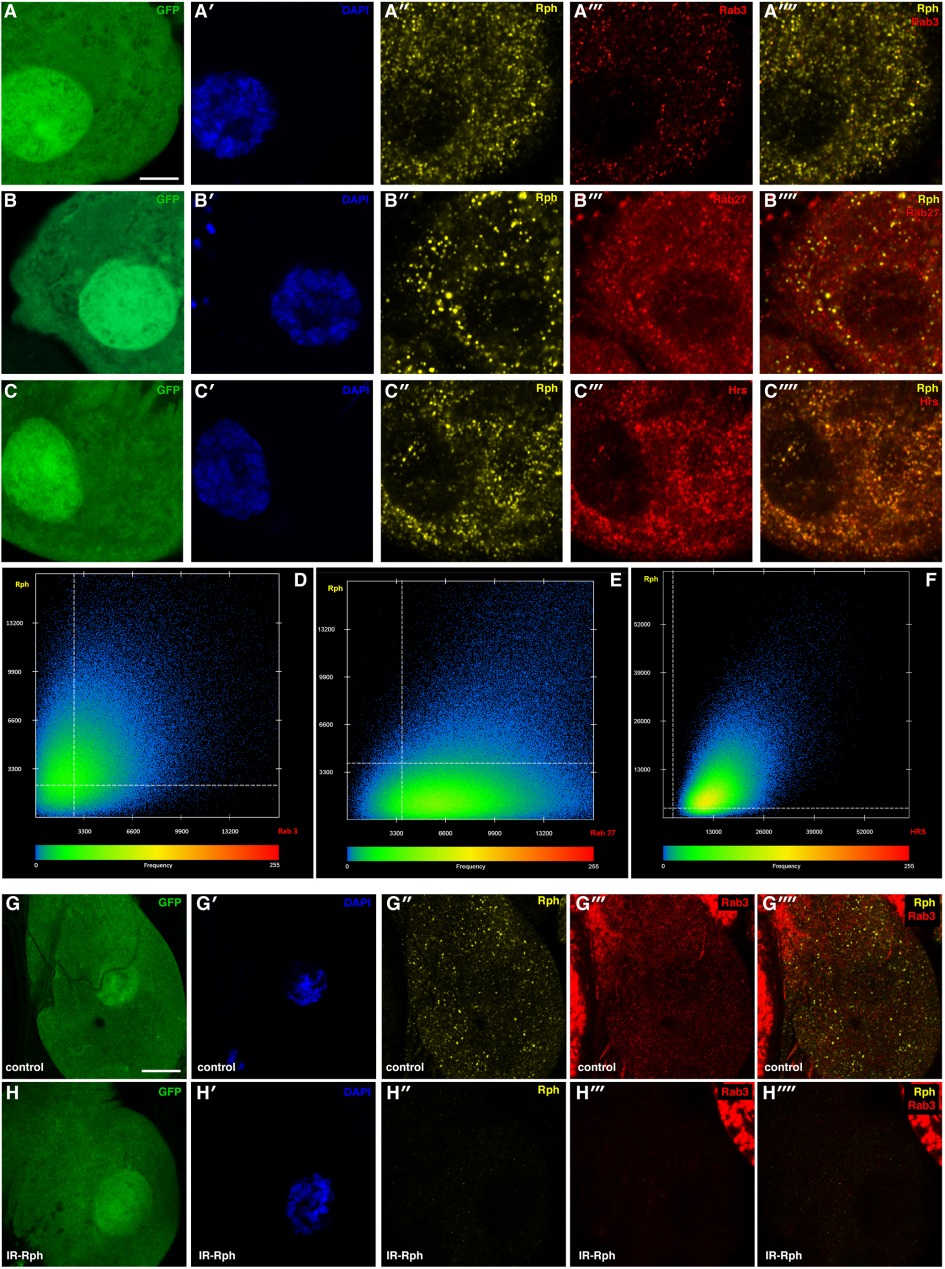
**The role of *Rph* in the endocytic pathway and vesicular transport. Silencing of *Rph* decreases Rab3^+^ vesicles.** (A-H) Double immunofluorescence with antibodies against Rph (yellow in A″,A⁗,B″,B⁗,C″,C⁗,G″,G⁗,H″ and H⁗), Rab3 (red in A‴,A⁗,G‴,G⁗,H‴ and H⁗), Rab27 (red in B‴ and B⁗) or Hrs (red in C‴ and C⁗) in control pericardial nephrocytes, *Hand-Gal4>UAS-GFP*, labelled with GFP (green in A,B,C,G,H) and DAPI (blue in A′, B′,C′,G and H). (D-F) Scatter plots showing the intensities of Rph signals plotted on the *y*-axis versus the intensities of Rab3 (D), Rab27 (E) and Hrs (F) on the *x*-axis. Threshold intensity levels on both axes are marked by discontinuous white lines in the three plots. The bar with the coloured pattern indicates the frequency of the pixels, with the red colour as the most frequent. Scale bars: 5 μm (A-C″″); 10 μm (G-H″″).

It is well known that Rph participates in vesicular trafficking in other cell types, such as neurons (Burns et al., 1998). It has been shown that Rph can interact with Rab3a and Rab27a *in vitro* and can make complexes with these Rabs in rat adrenal medulla PC12 cells (Fukuda et al., 2004). Moreover, previous studies in mammalian models found Rph3a in complex with Rab3a in the kidney, in which they specifically localize in podocytes (Rastaldi et al., 2003). Given that the main effects of Rph3a are mediated by these Rab proteins in neuronal and endocrine systems, we performed double immunofluorescence with antibodies against Rph and Rab3 or Rab27 in pericardial nephrocytes (Fig. 3). Image analysis of the signals revealed that 58.8% of the Rph signal overlapped with Rab3, whereas 80% of the Rab3 signal coincided with Rph (Fig. 3A-A⁗,D). In the case of the colocalization with Rab27, we observed that almost all the Rph signal colocalized with the Rab27 protein (more than 96% of colocalization percentage), whereas only 13.9% of the Rab27 signal was overlapped with Rph (Fig. 3B-B⁗,E). These data support that the protein interaction of mammalian Rph in vesicular transport is conserved by its *Drosophila* orthologue in nephrocytes.

To study the role of Rph in the endocytic pathway, we performed a double immunofluorescence assay against Rph and Hepatocyte growth factor-regulated tyrosine kinase substrate, Hrs, (Fig. 3C-C⁗) in pericardial nephrocytes. Hrs is an essential protein that has been implicated in cell signaling and intracellular membrane trafficking (Raiborg and Stenmark, 2002; Raiborg et al., 2001). Several reports have demonstrated a role for Hrs in endocytic sorting of ubiquitinated membrane protein (Raiborg and Stenmark, 2002). According to the colocalization coefficients, 100% of the Rph signal colocalized with Hrs and 98.9% of Hrs signal colocalized with Rph (Fig. 3C-C⁗,F). These results suggested a role for Rph in the pericardial nephrocyte endocytic pathway and vesicular trafficking.

To test the functional relevance of lack of *Rph* in intracellular membrane trafficking, we performed an immunofluorescence assay against Rab3, Rab27 and Hrs in *Rph* RNAi knockdown nephrocytes.

We observed that the Hrs signal decreased ∼40% compared to control nephrocytes (Fig. S6), Rab3 expression decreased robustly (up to 70%; Fig. 3G-H″″, Fig. S7), and Rab27 signal remained unchanged (Fig. S6). This result indicates that the loss of Rph has a strong impact in Rab3^+^ vesicles and suggests an alteration in endocytic trafficking.

**Fig. 4.**
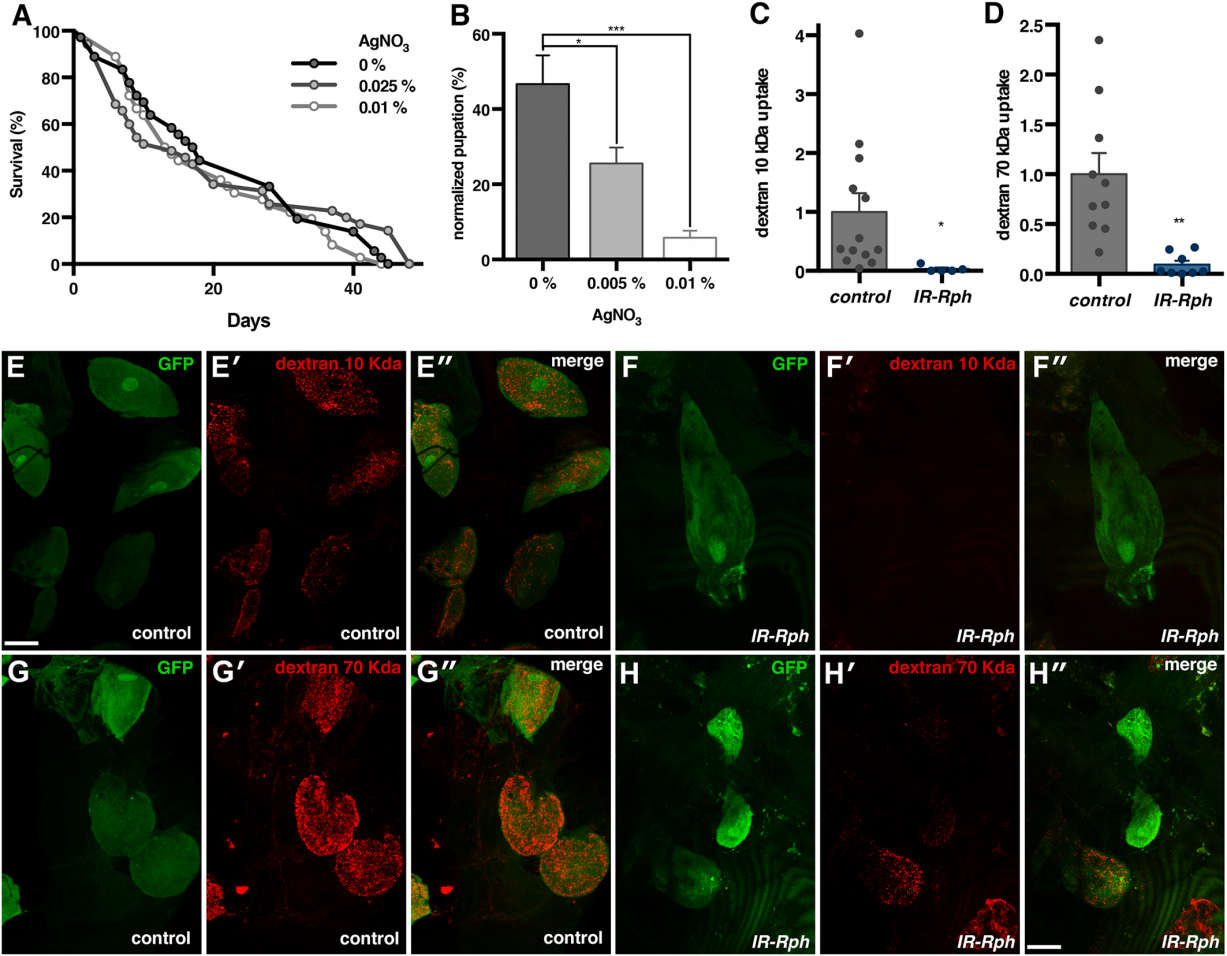
***Rph* is necessary for efficient molecular uptake in nephrocytes, *Rph* interference sensitizes larvae to AgNO_3_ toxicity.** (A) Survival curves of *Rph* RNAi knockdown flies (*Hand-Gal4>UAS-IR-Rph*) fed with standard (0%) or AgNO_3_-supplemented food at growing concentrations (0.005% and 0.01%) at 25°C, showed that the toxin did not affect the survival of adult *IR-Rph* flies [log-rank (Mantel–Cox) test]. (B) Pupation percentage of *IR-Rph* flies normalized to control flies (*Hand-Gal4>UAS-GFP*) was strongly reduced in larvae fed with 0.005% and 0.01% concentrations of AgNO_3_. (C-H) Dextran uptake analysis. Fluorescence images of the dextran uptake assay in control (*Hand-Gal>UAS-GFP* in E-E″ and G-G″) and *Rph* RNAi knockdown nephrocytes (*Hand-Gal4>UAS-GFP UAS-IR-Rph* in F-F″ and H-H″) performed by feeding the flies with fluorescently labelled dextran at either 10 kDa (red in E-E″ and F-F″) or 70 kDa (red in G-G″ and H-H″). Quantification of uptake efficiency in the indicated genotypes with 10 kDa (C) or 70 kDa (D) of dextran showed a clear decrease of dextran uptake in the *IR-Rph* nephrocytes. Individual data points are indicated. Data are mean±s.e.m. Two-tailed unpaired Student's *t*-test. *P<0.05, **P<0.01, ***P<0.001. Scale bars: 30 μm.

Nephrocyte scavenger activity of toxic molecules, such as ingested silver nitrate (AgNO_3_), is mediated by endocytosis, and disruption of this pathway increases the mortality of larvae fed with AgNO_3_ (Das et al., 2008). To test whether reduced *Rph* expression affects this crucial function of nephrocytes, we obtained the survival curves of adult and larval *Rph* RNAi knockdown flies fed with an AgNO_3_-supplemented diet. In adults, we observed a reduction in lifespan in the *Rph* RNAi knockdown flies (Fig. S3G) that was not worsened by AgNO_3_ (Fig. 4A). When larvae were fed with increasing concentrations of AgNO_3_, pupation percentage dropped in *IR-Rph* larvae relative to wild-type controls (Fig. 4B, Fig. S3H). These findings indicate that reduced levels of Rph in *Drosophila* sensitize larvae to the toxicity of AgNO_3_, which supports the notion that endocytosis is altered in *Rph* RNAi knockdown flies.

### Reduced *Rph* expression alters molecular reabsorption by pericardial nephrocytes

The insect nephrocyte combines filtration with protein reabsorption, using evolutionarily conserved genes and subcellular structures, which support its usefulness as a simplified model for both podocytes and the renal proximal tubule function (Fu et al., 2017a). Previous studies have shown that *Drosophila* nephrocytes can uptake fluorescent proteins from haemolymph (Weavers et al., 2009), which can be used as a reliable functional readout for nephrocytes *in vivo*. Circulating dextran molecules are filtrated from the haemolymph, endocytosed, and stored by nephrocytes, but the efficiency of this process is dependent on the size of the particles. In normal conditions, dextran molecules below 70 kDa easily cross the ND and are endocytosed in the labyrinthine channels by the *Cubn-Amnionless* system (Hermle et al., 2017; Zhang et al., 2013b). Defects in endocytosis might inhibit the uptake of proteins regardless of their size, and defects in the ND structure might facilitate the access of larger molecules to the labyrinthine channels (Hermle et al., 2017). Therefore, the accumulation of fluorescent dextran in the nephrocytes reflects both filtration efficiency and endocytosis. By adding fluorescently labelled dextrans of 10 kDa (Fig. 4E-E″,F-F″) and 70 kDa (Fig. 4G-G″,H-H″) to the dissected nephrocytes of adult flies, we observed that even the smallest sizes of dextran could not accumulate in the *Rph* RNAi knockdown nephrocytes (Fig. 4C,D). Thus, reduced expression of *Rph* alters molecular uptake by nephrocytes.

**Fig. 5.**
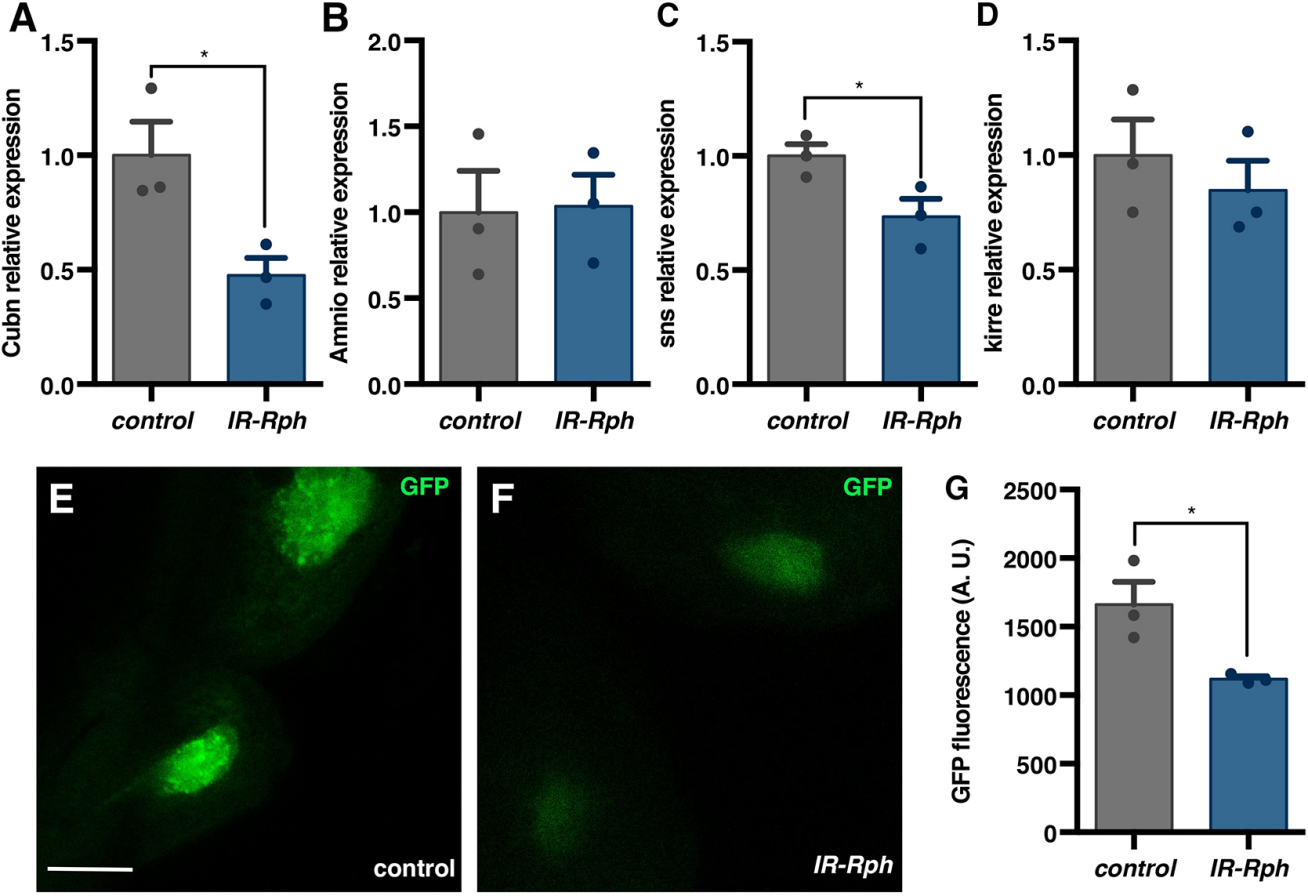
***Rph* RNAi knockdown downregulates genes implicated in the endocytic pathway.** (A-D) RT-qPCR to quantify expression levels of different nephrocyte-specific genes in the indicated genotypes (*Hand-Gal4>UAS-GFP* and *Hand-Gal4>UAS-GFP UAS-IR-Rph*) relative to endogenous controls. (E,F) Confocal images of adult control (*sns-Gal>UAS-GFP UAS-IR-bcd* in E) and *IR-Rph* (*sns-Gal4>UAS-GFP UAS-IR-Rph* in F) nephrocytes expressing GFP reporter (green) under the control of the *sns-Gal4* driver with the same settings. (G) Arbitrary units (A.U.) of fluorescence measured in control (*sns-Gal>UAS-GFP UAS-IR-bcd*) and *Rph* RNAi knockdown (*sns-Gal4>UAS-GFP UAS-IR-Rph*) flies. Individual data points are indicated. Data are mean±s.e.m. Two-tailed unpaired Student's *t*-test. *P<0.05. Scale bar: 10 μm.

*Drosophila Cubn* and *Amnionless* are specifically expressed in nephrocytes and function as co-receptors for protein and molecular uptake, similar to their roles in the mammalian proximal tubule (Hermle et al., 2017; Zhang et al., 2013b) and podocytes (Prabakaran et al., 2012; Gianesello et al., 2017). To test the hypothesis that small size molecules were not endocytosed owing to an alteration in the *Cubn-Amnionless* system, we performed a quantitative determination of *Cubn* and *Amnionless* expression. Quantification of the transcripts of these two genes showed a reduction of *Cubn* expression of ∼50% of normal levels in the dissected *Rph* RNAi knockdown nephrocytes, whereas *Amnionless* levels were normal (Fig. 5A,B). These data suggest a relevant role for *Rph* in protein reabsorption through the maintenance of *Cubn* expression.

The expression of *sns* and *kirre* transcripts, which encode proteins that form the slit pores, was measured in control and *IR-Rph* nephrocytes. Although there were no changes in the expression of *kirre* (Fig. 5D), *sns* expression was 20% lower in the *IR-Rph* flies compared to controls (Fig. 5C). To independently assess the reduction of *sns* expression, we used a *sns-Gal4* line to drive expression of the GFP protein as a reporter of *sns* expression. Semi-intact heart preparations of *sns-Gal4>UAS-GFP UAS-IR-Rph* flies were directly visualized under the fluorescence microscope (Fig. 5E,F), and homogenates were quantified for GFP fluorescence levels (Fig. 5G). We observed a decrease in the fluorescence signal in the *Rph* RNAi knockdown nephrocytes, thus supporting a role for Rph in the regulation of *sns* expression.

### *Rph* is required for maintaining labyrinthine channels of nephrocytes

**Fig. 6.**
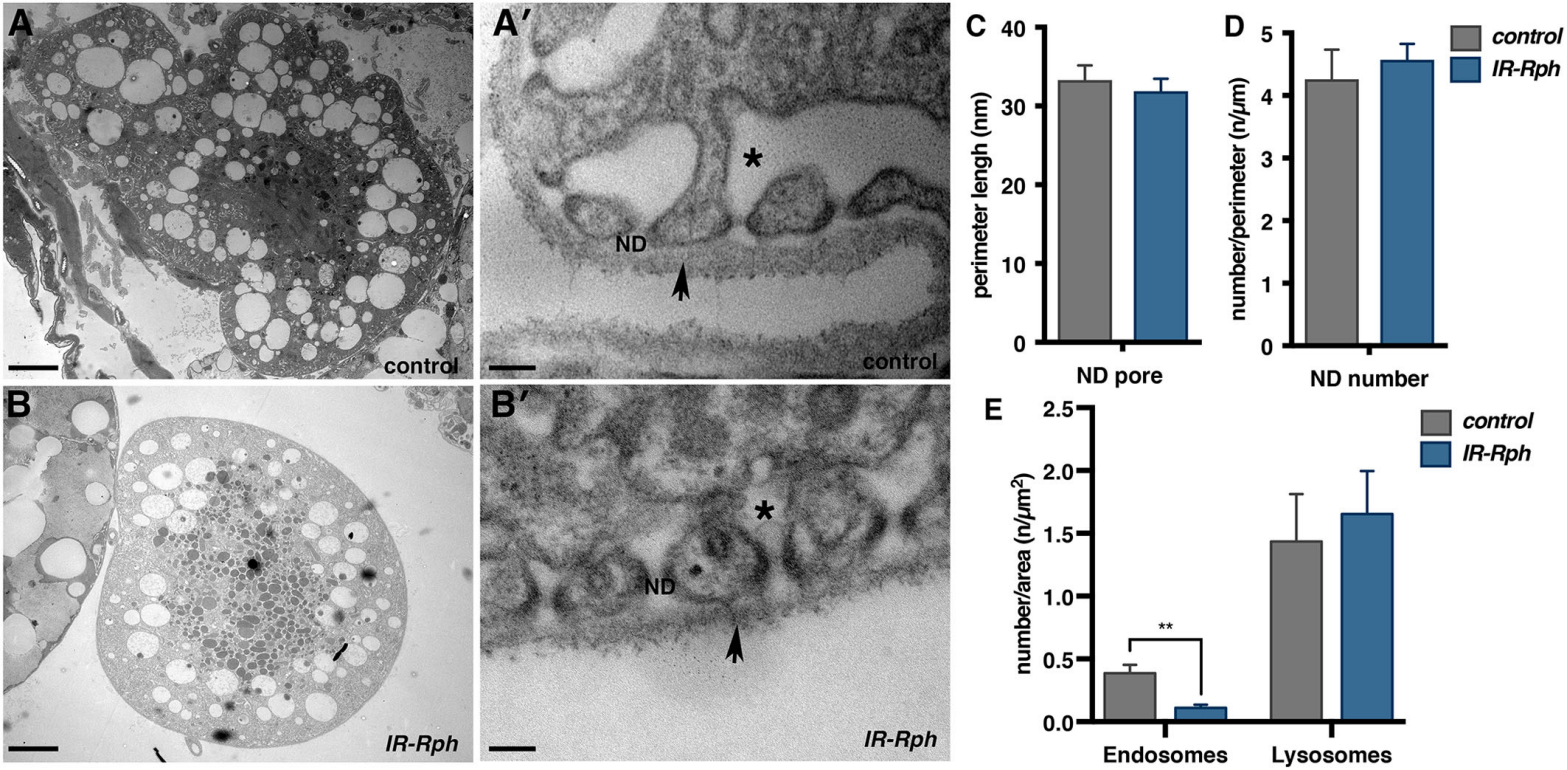
**Rph is essential for maintaining labyrinthine channels.** (A,B) In comparison with control flies (*Hand-Gal4>UAS-GFP* in A and A′), the *IR-Rph* nephrocytes (*Hand-Gal4>UAS-GFP UAS-IR-Rph* in B and B′) did not have labyrinthine channels (asterisk) and exhibited an altered basement membrane (arrow in A′ and B′). Scale bars: 2 μm (A,B); 100 nm (A′,B′). (C-E) Although the size of the pores (C), the number of ND per μm of nephrocyte perimeter (D), and the number of lysosomes (E) were the same, the number of endosomes (E) was reduced in *Rph* RNAi nephrocytes. Data are mean±s.e.m. Two-tailed unpaired Student's *t*-test. **P<0.01.

The filtration slit is a key functional structure in nephrocytes and disruption of the genes that form it leads to nephrocyte death (Zhuang et al., 2009). Transmission electron microscopy (TEM) was therefore used to examine the ultrastructure of pericardial nephrocytes in control and *IR-Rph* flies (Fig. 6). In comparison to control nephrocytes (Fig. 6A,A′), the size and number of NDs were not altered in the nephrocytes of the *IR*-*Rph* flies (Fig. 6B-D). However, the labyrinthine channels were absent in the nephrocytes of these flies and the basement membrane was altered in some regions. In addition, the number of endocytic vesicles and vacuoles was significantly reduced (Fig. 6E, Fig. S8), whereas there were no changes in the number of lysosomes (Fig. 6E). In agreement with our results regarding dextran uptake impairment and AgNO_3_ sensitization, the changes in nephrocyte ultrastructure support disruption of the endocytic trafficking in the *Rph* RNAi knockdown nephrocytes.

### RA rescues the filtration and the endocytosis defect caused by the interference of *Rph* expression

**Fig. 7.**
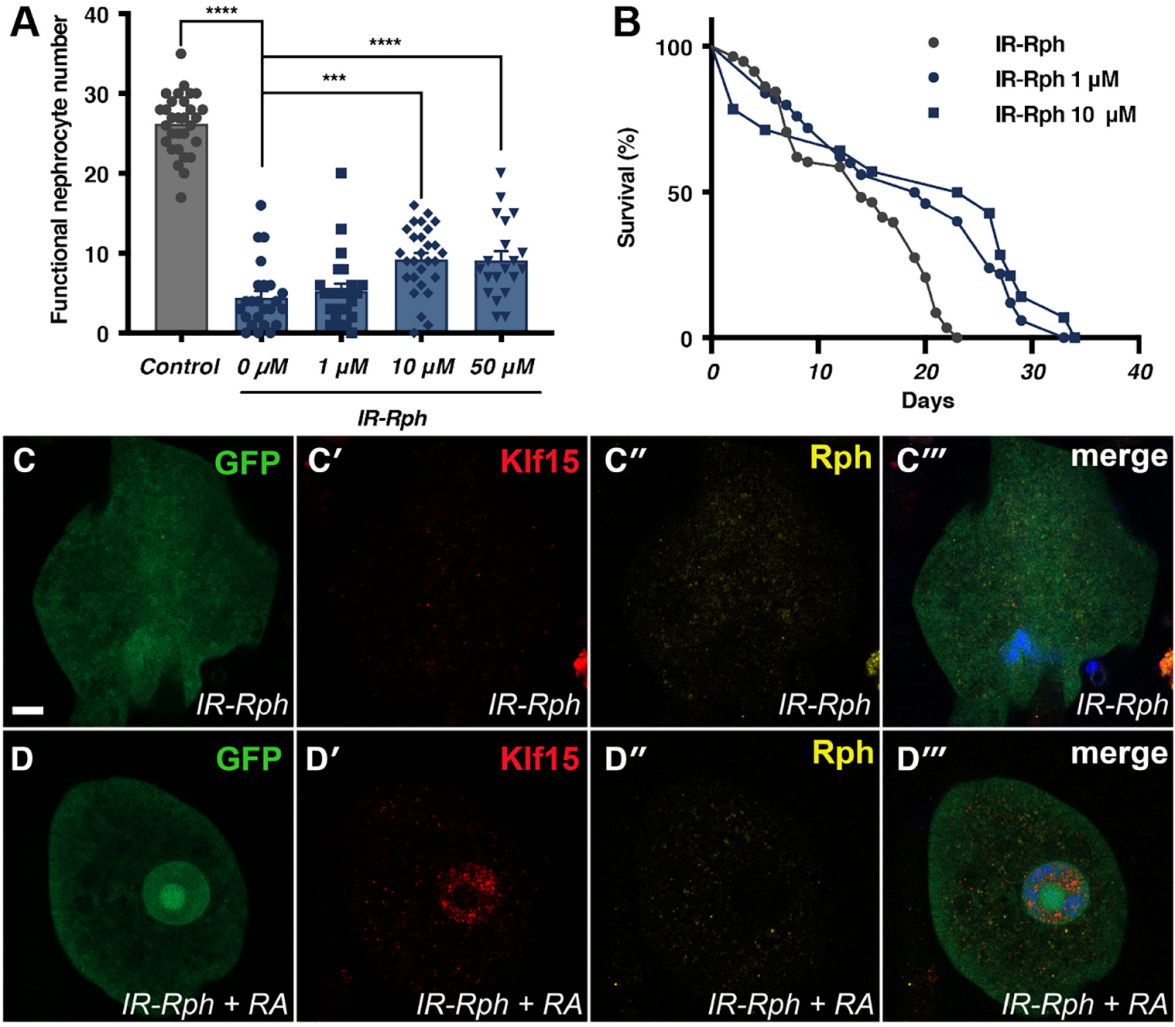
**RA rescues functional nephrocyte number, survival and *Klf15* expression of *IR- Rph* flies.** (A) Functional nephrocyte number of 1-week-old *IR-Rph* flies at different concentrations of RA (1 μM, 10 μM, and 50 μM). Data are mean±s.e.m. Two-tailed unpaired Student's *t*-test, *n*≥20 flies, ***P<0.001, ****P<0.0001. (B) Survival curves of *IR-Rph* flies fed with standard nutritive media (*IR-Rph*, black line) or supplemented with RA at 1 μM or 10 μM (*IR-Rph* with 1 μM and *IR-Rph* with 10 μM, blue lines) [log-rank (Mantel–Cox) test]. Individual data points are indicated in A and B. (C,D) Representative confocal images of pericardial nephrocytes expressing GFP reporter (green in C,C‴,D, D‴) and *UAS-IR-Rph* under the control of the *Hand-Gal4* driver. Immunostaining with anti-Klf15 antibody (red in C′,C‴,D′,D‴) revealed a higher signal of Klf15 in nephrocytes treated with 10 μM of RA (*IR-Rph*+RA, D′,D‴) than in the control (*IR-Rph*, C′,C‴). RA did not have any effect on Rph signal in both non-treated and RA-treated nephrocytes (C″,C‴,D″,D‴) that expressed *IR-Rph* construct. Nuclei were counterstained with DAPI (blue). Scale bar: 10 μm.

RA is a metabolite of vitamin A, which is acquired directly through the diet as it cannot be synthesized by the body. It is involved in different processes such as cellular differentiation, proliferation, apoptosis regulation and inflammation inhibition (Niederreither and Dollé, 2008). It is known that RA also plays a role in podocyte differentiation via *KLF15* regulation (Mallipattu et al., 2012; Niederreither and Dollé, 2008; Guo et al., 2018; Mallipattu and He, 2015; Sharma et al., 2014). The restoration of podocyte differentiation markers by RA has allowed this metabolite to be considered as a treatment for renal diseases (Mallipattu and He, 2015). Non-functional pericardial nephrocytes are characterized by a loss of Sns functionality, including filtration and reabsorption defects. These non-functional nephrocytes undergo the process of dedifferentiation, and their nuclei are fragmented and their GFP signal is reduced (Fig. 1B-B″). The expression of the *IR-Rph* construct causes a loss of function in the nephrocytes and, consequently, a decrease in their number and a decrease in fly survival. In this study, we administered different concentrations of RA to *IR-Rph* flies. We observed that the flies treated with RA presented a significantly higher number of functional nephrocytes than the *IR-Rph* flies fed with the standard nutritive media (Fig. 7A). Additionally, these RA-treated flies showed significantly longer survival curves (Fig. 7B). These results suggest that RA could improve functional defects in *IR-Rph* flies.

In order to determine whether this functional restoration is due to dedifferentiation inhibition, as described in podocytes via the regulation of *KLF15* (Mallipattu et al., 2016, 2012), We carried out an immunoassay for these proteins. In *IR-Rph* flies fed with nutritive medium containing RA at 10 μM (Fig. 7D-D‴), the Klf15 signal was clearly more intense than in *IR-Rph* flies fed with standard nutritive medium (Fig. 7C-C‴), whereas in both cases Rph levels in the nephrocytes were reduced compared with control (healthy) nephrocytes (compare nephrocytes from Fig. 7C″,D″ with the nephrocyte from Fig. 1A″) and RA had no effect on Rph levels in *IR-Rph* flies fed with the compound (Fig. 7C″,D″). These results indicate that the increment in the functional nephrocyte number and the lifespan of the flies could be a result of *Klf15* expression regulation.

**Fig. 8.**
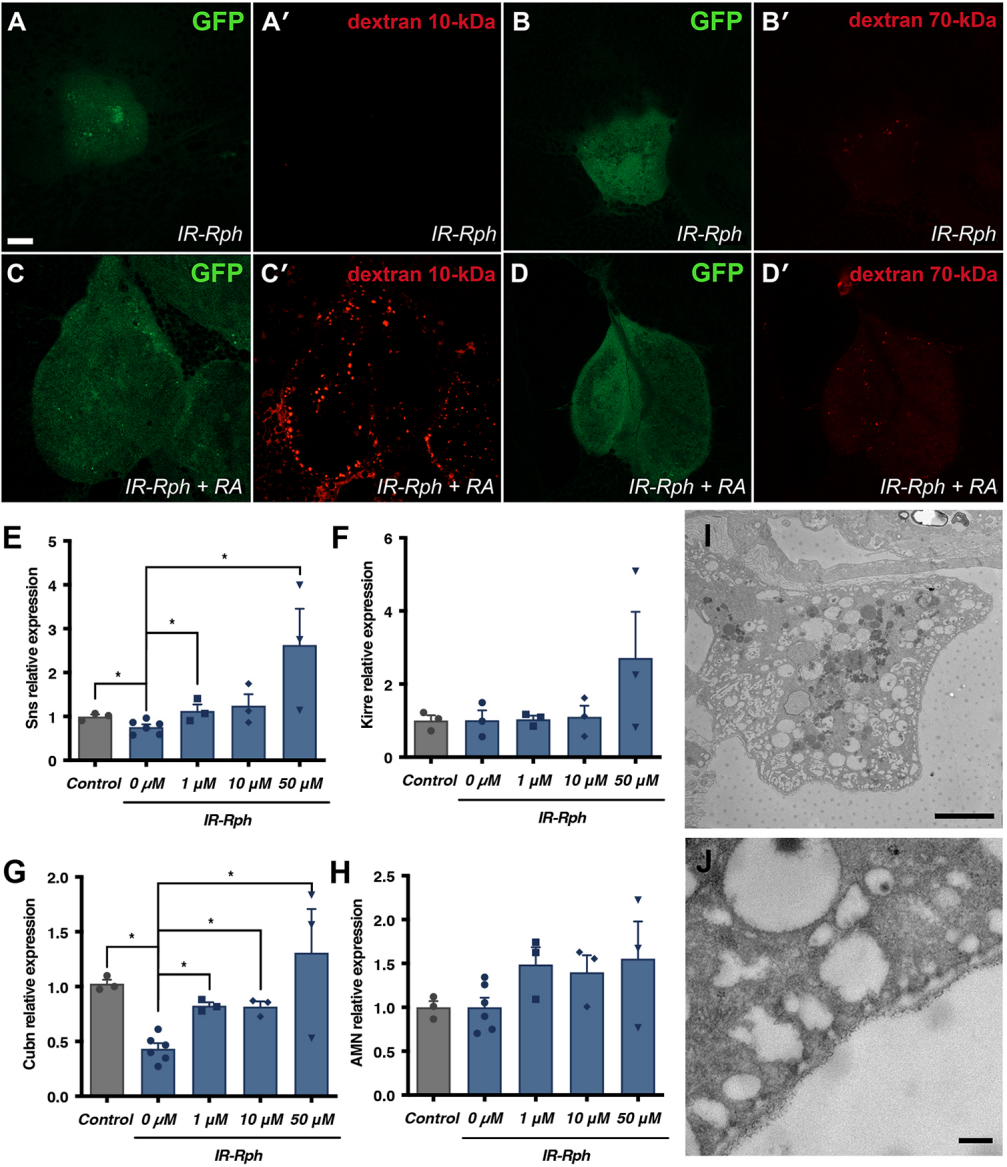
**RA rescues the nephrocyte filtration process and endocytosis.** (A-D) Representative confocal images of pericardial nephrocytes expressing GFP reporter (green in A,B,C,D) and *UAS-IR-Rph* under the control of the *Hand-Gal4* driver. 10-kDa and 70-kDa dextran uptake assay in *IR-Rph* flies fed with standard media (A,A′,B,B′) or supplemented with 10 μM of RA (C,C′,D,D′). (E-H) RT-qPCR analysis quantifying expression levels of different nephrocyte-specific genes in the indicated genotypes (*Hand-Gal4>UAS- GFP*, control; and *Hand-Gal4>UAS-GFP UAS-IR-Rph*) relative to endogenous controls. (I,J) TEM images of *IR-Rph* nephrocytes treated with 10 μM of RA. Individual data points are indicated in E-H. Data are mean±s.e.m. Two-tailed unpaired Student's *t*-test. *P<0.05. Scale bars: 10 μm (A-D′); 4 μm (I); 200 nm (J).

We assessed whether RA was capable of rescuing reabsorption or endocytosis by administering 10 kDa and 70 kDa of dextran to *IR-Rph* flies fed with either standard nutritive medium (control) or supplemented with RA at 10 μM (Fig. 8). Flies treated with RA captured the 10-kDa dextran molecules significantly more efficiently than the non-treated group (Fig. 8A,A′,C,C′). The 70-kDa dextran molecules were also captured (Fig. 8B,B′,D,D′), however not as efficiently as in the control nephrocytes shown in Fig. 6. These findings suggest that RA partially rescues the filtration and endocytic processes in *Drosophila* nephrocytes.

Accordingly, when RA was administered, *sns* and *Cubn* expression both increased to normal levels in *IR-Rph* flies (Fig. 8E,G), with no changes in *Kirre* and *Amnionless* gene expression (Fig. 8F,H). These findings suggest that the compound rescues the filtration and endocytic functions mediated by the Cubn/Amnionless complex, which were decreased in *IR-Rph* flies. This restoration could be due to the upregulation of *Klf15*, which controls *sns* expression, as proven in previous studies (Mallipattu et al., 2012; Guo et al., 2018). Additionally, as shown in the TEM images, the nephrocytes of RA-treated flies recovered the labyrinthine channel structures (Fig. 8I,J), which were absent in non-treated *IR-Rph* nephrocytes (Fig. 6). These results suggest that the restoration of *Klf15* expression by RA is sufficient to partially rescue the filtration and endocytosis defects shown by *IR-Rph* flies.

## DISCUSSION

*Rph3A* expression is known to be altered in proteinuric diseases, such as in human and mouse models (Rastaldi et al., 2003). In addition, previous studies have suggested that a polymorphism in the *RPH3A* gene increases the risk of microalbuminuria (Marrachelli et al., 2014). However, little is known about the role of this protein in podocytes. Taking advantage of the high degree of conservation of the filtration structures between human podocytes and *Drosophila* nephrocytes, and the myriad of genetic tools available in flies, we studied the role of *Rph* in this model system and the consequences of silencing its expression.

In this study, we show that Rph, a protein that is well known as a Rabs effector, is expressed in *Drosophila* pericardial nephrocytes, and has a relevant function in haemolymph clearance, the maintenance of essential structures and nephrocyte cell fate. However, it is not clear whether the dysfunction of one of these aspects is a cause or consequence of the dysfunction of the others. As Fig. S9 illustrates, the loss of Rph function had an impact on basement membrane thickness and the maintenance of labyrinthine channels. Moreover, Rab3^+^ vesicles were greatly reduced in *Rph* RNAi knockdown nephrocytes, indicating a vesicular trafficking reduction and suggesting a blockage of Rab3-mediated endocytosis. Consequently, reduced levels of *Rph* in *Drosophila* nephrocytes sensitized larvae to the toxicity of AgNO_3_. Of note, we did not observe toxicity in adult flies, which supports the hypothesis that nephrocytes are the primary system for mitigating xenotoxin insults in larvae, but other cells can compensate for their loss in adults (Ivy et al., 2015). Finally, the interruption of the endocytosis pathway suggests a role for *Rph* in protein reabsorption, presumably by the modulation of *Cubilin* expression, which was reduced.

Several studies show that the endocytic pathway plays an important role in the development, maintenance, and damage of the podocyte and might lead to alterations in cell morphology (Hochapfel et al., 2017; Bechtel et al., 2013; Chen et al., 2013). In connection with this observation, our data support a role for *Rph* in the maintenance of nephrocytes, which was assessed by the reduced expression of terminal marker *Klf15*. It is known that reduced levels of KLF15 in podocytes promote alteration of the filtration and endocytic pathways, which ultimately causes the loss of cell fate (Mallipattu et al., 2012). In our study, Klf15 levels were reduced by the interference of *Rph* expression but either those levels were not low enough to produce changes in *Amnionless* expression or we could not detect Amnionless reduction with RT-qPCR. In this regard, it should be taken into account that the dissected tissue sample used for RNA extraction to perform RT-qPCR did not exclusively contain nephrocytes. It was contaminated with remnants of cardiac tissue and body wall muscles. This experimental limitation might have concealed small differences in gene expression levels. Of note, we were able to detect an impact of Klf15 levels on *Cubilin* expression, which is part of the Cubn-Amnionless complex.

A reduction in the levels of *Rph* also affects the expression of genes directly involved in the nephrocyte structure. Sns and Kirre (human Nephrin and NEPH1 orthologues) interact through their extracellular domains to form the ND and are essential for its formation. The *Cubn-Amnionless* system is in charge of the endocytosis of small size proteins across the labyrinthine channels (Hermle et al., 2017; Zhang et al., 2013b). Both systems can be altered by a reduction in the gene expression or mislocalization of any of their components.

The absence of *sns* expression led to a dramatic reduction of ND number (Zhuang et al., 2009). In our case, TEM images did not show any effect in ND number, probably because the reduction in *sns* expression of *IR-Rph* nephrocytes was only 20%. Accordingly, recent studies have documented the significance of cytoplasmic Sns in regulating actin organization (Muraleedharan et al., 2016), which could be direct or a result of altered cortical actin when ND proteins are lost or reduced. The same study highlights the role of the reciprocal regulation between cytoskeletal components and ND proteins as essential for size and charge-dependent filtration.

The RA-treated nephrocytes showed *sns* and *Cubn* expression levels similar to control nephrocytes (Fig. 8E,G), presented labyrinthine channels (Fig. 8I) and were able to carry out the filtration function (Fig. 8C′) and partial endocytosis (Fig. 8D′). These restorations in *IR-Rph* nephrocytes are probably due to the upregulation of *Klf15* expression through the administration of RA and demonstrate that forced maintenance of the nephrocyte differentiation state rescues Rph silencing phenotypes.

Our data highlight the relevance of *Rph* in nephrocyte structure and function. Specifically, knockdown of *Rph* seems to impair nephrocyte performance at two levels: filtration by basement membrane disruption and subsequent cytoskeletal modifications leading to important structural alterations (absence of labyrinthine channels) and nephrocyte loss, and protein uptake by influencing *Cubn* expression and vesicular trafficking (Fig. S9). This suggests a dual role for *Rph3A* in the mammalian excretory system and could explain why a polymorphism in human RPH3A leads to microalbuminuria (Marrachelli et al., 2014). Importantly, the *Rph3A* expression pattern in mammals is not well described, and its presence in different tissues, either podocyte or proximal tubule, might influence its activity. The role of Rph in the maintenance of the structure and function of nephrocytes opens a window to explore the contribution of *RPH3A* gene polymorphisms to the risk of developing chronic kidney disease.

## MATERIALS AND METHODS

### *Drosophila* strains

*Hand-Gal4, UAS-IR-Rph* (BDSC, 25950), *UAS-IR-bcd*, *UAS-GFP* and *yw* were obtained from BDSC (Indiana University), s*ns-Gal4 UAS-GFP* was obtained from M Ruiz-Gómez (Centro de Biología Molecular Severo Ochoa, Spanish National Research Council and Autonomous University of Madrid, Spain). Other *UAS-IR-Rph* lines used were line 1, 524338 (construct ID: 7330), and line 2, 109337 (construct ID: 107492), which were obtained from the Vienna *Drosophila* Resource Center. The recombinant line *Hand-Gal4 UAS-GFP* was generated during this study to mark adult pericardial nephrocytes. All crosses were maintained at 25°C on standard nutritive medium.

### *Drosophila* lifespan analyses

More than 50 newly hatched males per genotype were collected, placed in tubes containing standard nutritive medium or medium supplemented with different concentrations of AgNO_3_ (0.005% or 0.01% AgNO_3_) and kept at 25°C (Fig. 4A) or 29°C (Fig. S3G). The number of deceased flies was scored on a daily basis, and flies were transferred to fresh medium every 2 to 3 days. Survival curves were obtained using the Kaplan–Meier method, and statistical curve comparisons were carried out according to the log-rank (Mantel–Cox) test (α=0.05).

### Toxin stress assay

Twenty first instar larvae (L1) from each genotype were transferred to vials supplemented with different concentrations of AgNO_3_ and maintained at 25°C. The number of larvae that reached pupal stage was scored to determine the toxicity. Results were analyzed by two-tailed unpaired Student's *t*-test (α=0.05), applying Welch's correction whenever necessary. The experiment was performed in triplicate.

### Treatment with RA

One-day-old *Hand-GFP>UAS-IR-Rph* females were fed with standard media supplemented with different concentrations of RA diluted in food (1 μM, 10 μM and 50 μM) for 1 week; for the survival analyses, the flies were fed with this medium until their death, and for the Klf15 analysis, flies were fed with 10 μM RA from the larval stage to 1-week-old adults. RA-treated flies were maintained at 25°C for all tests, except for the lifespan analysis in which they were maintained at 29°C. All the experiments were performed in triplicate.

### Immunofluorescence staining

Adult hearts with pericardial nephrocytes from 7-day-old females were dissected in artificial *Drosophila* haemolymph according to Selma-Soriano et al. (2018a), fixed with 4% paraformaldehyde (PFA) in PBS for 20 min and permeabilized by washing three times with PBS containing 0.3% Triton X-100 (PBS-T) for 10 min. Then, hearts were blocked in PBS-T containing 0.5% bovine serum albumin for 30 min at room temperature and incubated with the corresponding primary antibody (1:100) overnight at 4°C. Primary antibodies used were anti-Rph (Abcam, ab3338; with 50% of homology), anti-Hrs (DSHB, AB 2722114), anti-Rab3 and anti-Rab27 (BD Bioscience, 610379 and 558532, respectively) and anti-Klf15 (Ivy et al., 2015) (Abcam, ab22851; with 59% of homology). After three washes with PBS-T, the secondary antibodies (1:200), AlexaFluor-647 donkey anti-rabbit (Life Technologies, A31573), anti-mouse biotinylated (Sigma-Aldrich, B7264) and anti-rabbit biotinylated (Sigma-Aldrich, B8895) were incubated for 2 h at room temperature. Hearts with pericardial nephrocytes were then incubated with ABC solution (ABC kit, VECTASTAIN) for 30 min at room temperature, followed by washes and incubation with streptavidin-Texas Red (Vector Laboratories, 1:500). All images were taken using an LSM 800 confocal microscope (Zeiss) and were processed using ZEN software.

### Rph, Klf15 and GFP signal quantification

ZEN software was used to quantify Rph, Klf15 and GFP signals from immunostaining images. The area of the nephrocyte signal was selected and the intensity of the pixels was scored. For the analysis, at least three different biological samples were used. Results were analyzed using a two-tailed unpaired Student's *t*-test (α=0.05), applying Welch's correction whenever necessary.

### *In vivo* GFP quantification

Homogenates from five 1-week-old females, per triplicate, were quantified using a fluorescence microplate reader (Tecan). Results were analyzed using a two-tailed unpaired Student's *t*-test (α=0.05), applying Welch's correction whenever necessary.

### Quantification of volume and number of nephrocytes

For the analysis of the number and volume of nephrocytes, adult female fly hearts were dissected in 1× PBS. Confocal images were obtained with a FLUOVIEW FV1000 confocal microscope (Olympus) using a 10× or 20× air objective. Three-dimensional structures were reconstructed from confocal stacks using Imaris 7.1 software (Bitplane). For quantification, confocal imaging settings were invariant within each experiment. For volume quantification, at least three nephrocytes from three different flies were analyzed.

### TEM

Dissected abdomens from 7-day-old females were processed as described previously by Selma-Soriano et al. (2018b). Briefly, abdomens were fixed in 1% PFA and 2% glutaraldehyde in 0.2 M phosphate buffer for 30 min and postfixed with 1% osmium tetroxide for 2 h. After dehydration, abdomens were embedded in epoxy resin. Semi-thin (1.5 μm) and ultra-thin sections were obtained using an ultramicrotome (Ultracut E, Reichert-Jung and Leica). Samples were analyzed using a JEOL JEM-1010 TEM at an operating voltage of 80 kV.

### Quantification of ultrastructure in TEM images

Quantification of TEM images was carried out by counting the number of SDs in the plasma membrane of pericardial nephrocytes at 8000× magnification. The pore size was measured at three different levels (innermost, middle and uppermost pore site) at 30,000× magnification. The number of endosomes and lysosomes was obtained from 800× magnification images of sections containing entire cells. The endosomes are the white vesicles near the membrane (Fig. S8) and the lysosomes are the dark vesicles located at the centre of the nephrocyte. Images from at least four different nephrocytes of the same genotype were scored. Results were analyzed using a two-tailed unpaired Student's *t*-test (α=0.05).

### *In vivo* nephrocyte filtration assay or dextran uptake assay

Adult female fly hearts were dissected in artificial *Drosophila* haemolymph and were incubated with 0.1 mg/ml 10-kDa dextran (AlexaFluor568, Invitrogen) or with 0.1 mg/ml 70-kDa dextran (Thermo Fisher Scientific, D1830). After 15 min of incubation, the hearts were washed three times with PBS. Pericardial nephrocytes were then immediately mounted for confocal imaging. Zen Software was used to measure the volume and signal of fluorescent dextran for the dextran quantification. At least three nephrocytes from three different flies were analyzed. Results were analyzed using a two-tailed unpaired Student's *t*-test (α=0.05).

### Real-time RT-qPCR

For RT-qPCR, RNA was first isolated using TRIzol Reagent (Invitrogen) from 100 dissected adult hearts (pooled). RNA purity and concentration were determined using a NanoDrop 1000 (Thermo Scientific). Total RNA (1 μg) was reverse transcribed using Superscript II Reverse Transcriptase (Invitrogen). SYBR-Green-based real-time qPCR was performed using a QuantStudio 5 Real-Time PCR System (Applied Biosystems). *Rp49* and *Tub48B* were used as endogenous references. In all cases, relative expression to the endogenous genes and the control group was obtained by the 2^−ΔΔCt^ method. Pairs of samples were compared using a two-tailed unpaired Student's *t*-test (α=0.05), applying Welch's correction whenever necessary.

## Supplementary Material

Supplementary information
