# Supplementary material for: Rabphilin involvement in filtration and molecular uptake in Drosophila nephrocytes suggests a similar role in human podocytes
Source: Dis Model Mech. 2020 Sep 21;13(9):dmm041509. doi: 10.1242/dmm.041509 (PMC7522021; doi:10.1242/dmm.041509)
Supplement: Supplementary information [file dmm-13-041509-s1.pdf]

Supplementary Figures

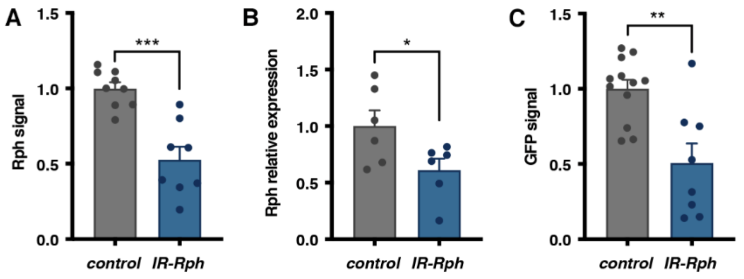

Figure S1. Rph and GFP levels are decreased in adult *IR-Rph Drosophila* pericardial nephrocytes. Relative quantification of Rph signal from immunofluorescence assay of control and *IR-Rph* nephrocytes (A). Reverse transcription-quantitative polymerase chain reaction to quantify *Rph* expression level in *Hand-Gal4>UAS-GFP* (control) and *Hand- Gal4>UAS-GFP UAS-IR-Rph* (*IR-Rph*) relative to endogenous controls (B). Relative quantification of GFP signal from immunofluorescence assay of control and *IR-Rph* nephrocytes (C). Student´s t-test. **p<0.001.

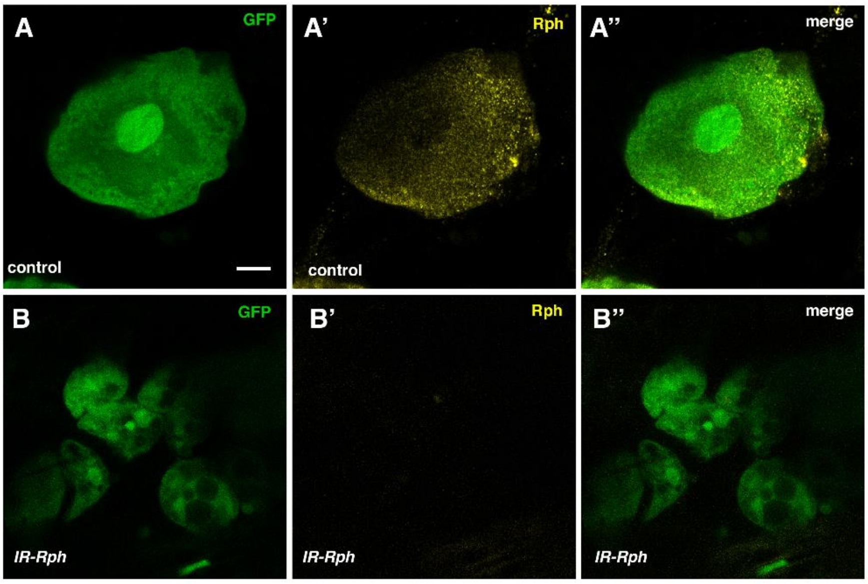

Figure S2. *Rph* is expressed in pericardial nephrocytes of *Drosophila* larvae. Representative confocal images of larvae expressing the GFP reporter (green in A, A'', B and B'') under the control of the *Hand-Gal4* driver. Immunostaining with anti-Rph antibody (A', A'', B' and B'') revealed Rph presence in pericardial nephrocytes of control larvae (A-A''), but not in larvae expressing a *Rph* RNA interference construct (B-B''). Scale bar = 10 µm.

Disease Models & Mechanisms • Supplementary information

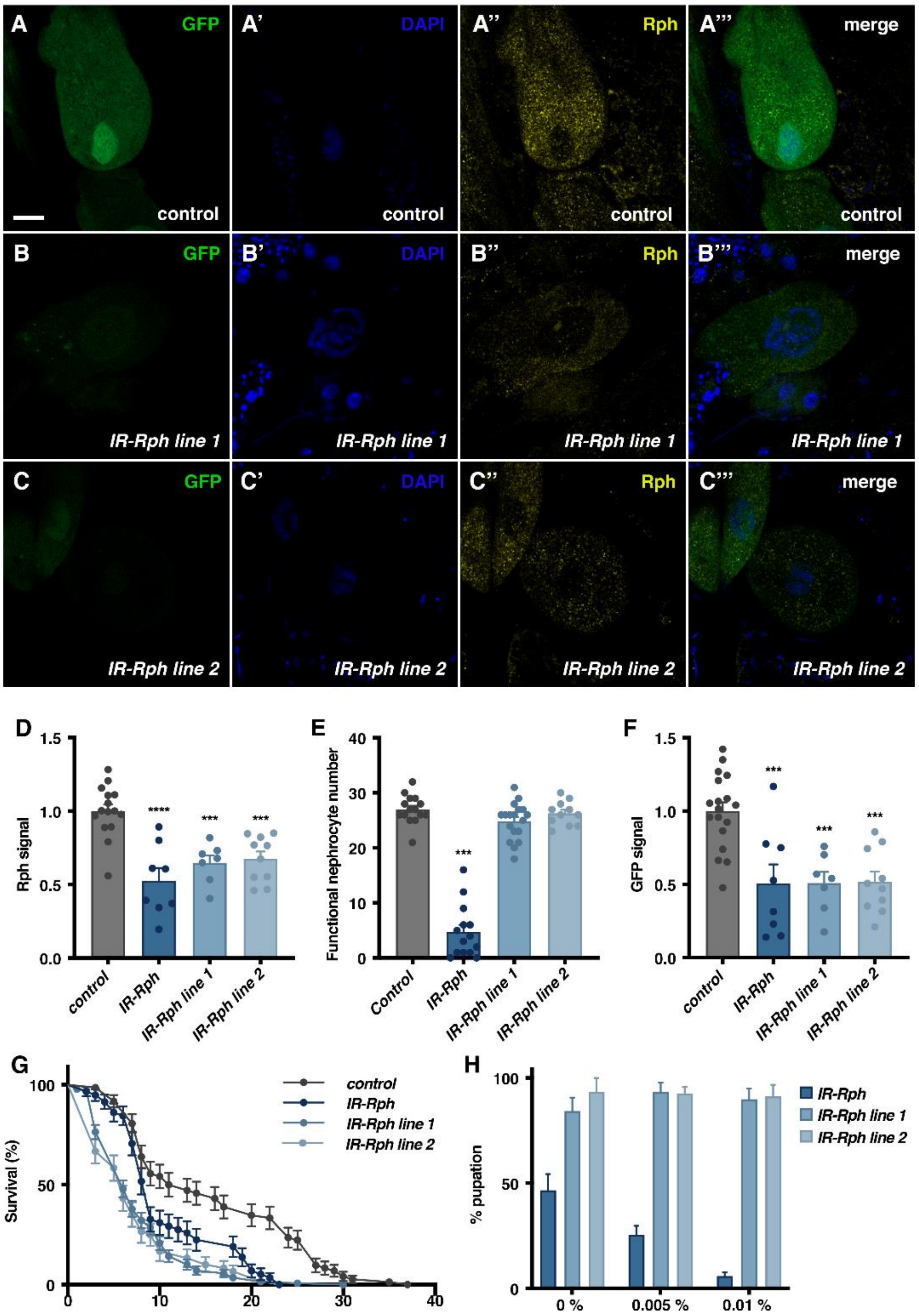

Figure S3. Other *Drosophila* lines with *Rph* interference expression in nephrocytes had reduced functional nephrocyte numbers and survival. Representative confocal images of adult flies expressing the GFP reporter (green in A, B, and C) under the control of the *Hand-Gal4* driver. Immunostaining with anti-*Rph* antibody (A´´, A´´´, B´´, B´´´, C'' and C''') revealed Rph presence in pericardial nephrocytes of control flies (A-A´´´), but this signal was reduced in flies expressing *Rph* RNA interference construct (B-B´´´ and C-C'''). Nuclei were counterstained with DAPI (blue in A', B' and C'). Scale bar = 10 µm. Quantification of Rph signal form immunostaining confocal images (D). Average number of functional pericardial nephrocytes (E). Quantification of GFP signal from immunostaining confocal images (F). Survival curves of control (Hand-Gal4>UAS-GFP) and *Rph* RNAi knockdown flies (*Hand-Gal4> UAS-IR-Rph*) fed with standard food, performed at 29 ºC (log-rank (Mantel-Cox) test) (G). Pupation percentage of *IR-Rph* larvae fed with 0.005 % and 0.01 % concentrations of $AgNO_3$ (H). ANOVA's test applying

Dunnet correction when it was necessary. *p<0.05, **p<0.01, ***p<0.001, ****p<0.0001.

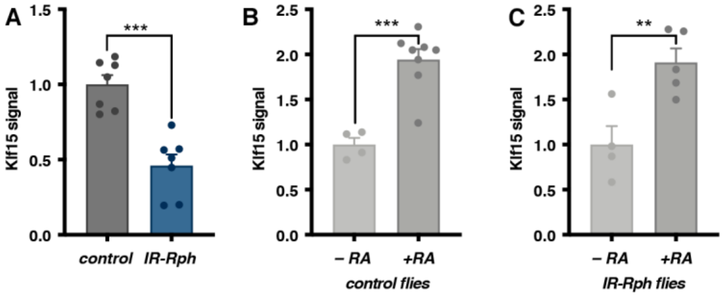

Figure S4. *Rph* interference expression decreased Klf15 levels in *Drosophila* pericardial nephrocytes. Quantification of Klf15 signal from immunostaining images from control and *IR-Rph* flies fed with standard nutritive media (A) and treated with 10 µM retinoic acid concentration in food (B and C).

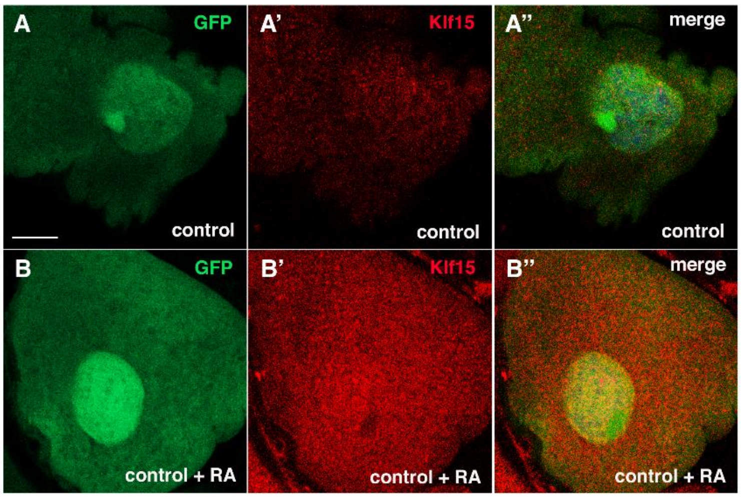

Figure S5. Retinoic acid increases *Klf15* expression in pericardial nephrocytes. Fluorescence microscopy images of control pericardial nephrocytes fed with standard nutritive media (A-A'') or supplemented with retinoic acid (B-B'') marked with GFP (green) and immunostained with an anti-Klf15 antibody (red). The signal of Klf15 is increased by the addition of retinoic acid. Scale bar =5 µm.

Disease Models & Mechanisms • Supplementary information

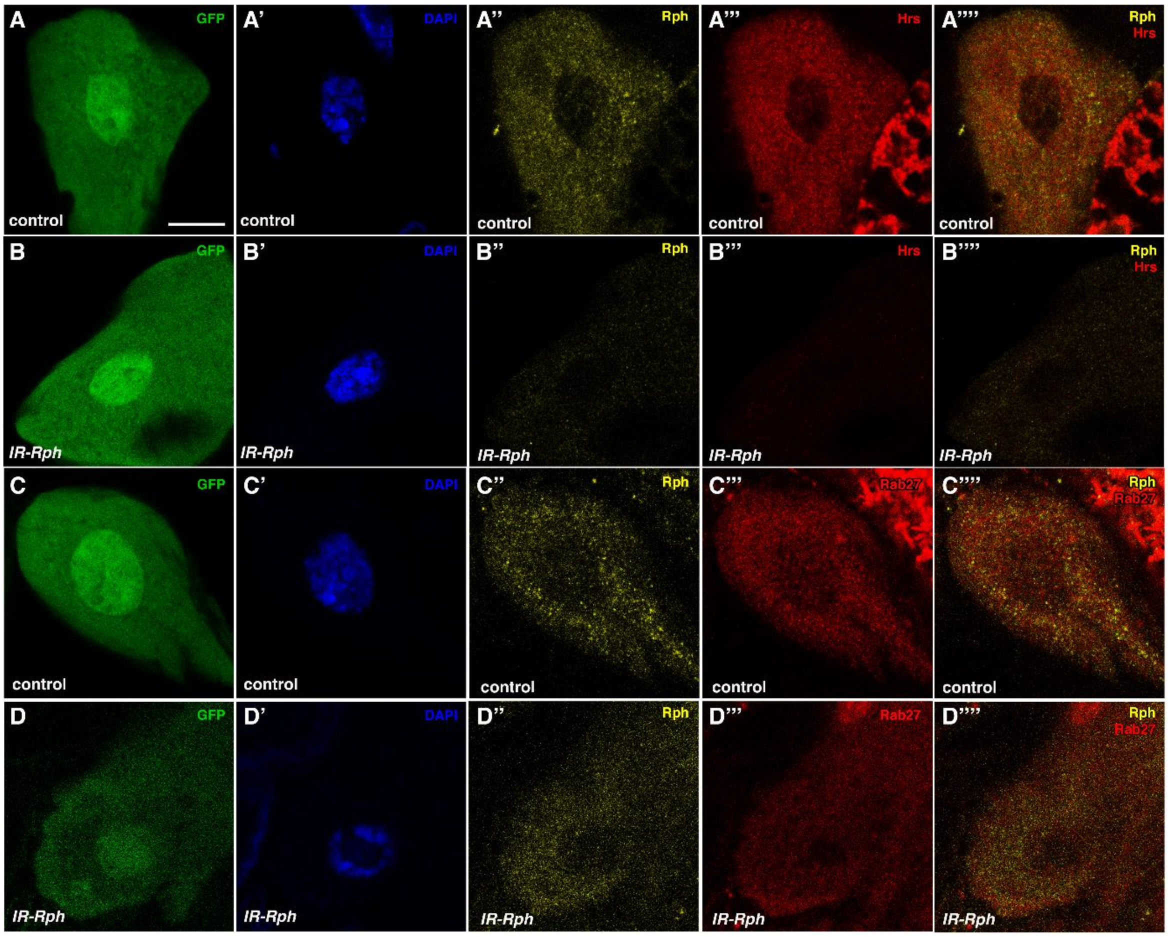

Figure S6. Downregulation of *Rph* expression decreases Hrs signal but it does not have effect in Rab 27 signal. Double immunofluorescence with antibodies against Rph (yellow in A’’, A’’’’, B’’, B’’’, C’’, C’’’’, D’’, and D’’’’) and Hrs (red in A’’’, A’’’’, B’’’, and B’’’’) or Rab 27 (red in C’’’, C’’’’, D’’’, and D’’’’) in control (*Hand-Gal4> UAS-GFP yw)* and *IR-Rph* pericardial nephrocytes (*Hand-Gal4>* UAS-GFP UAS-IR-Rph) marked with GFP (green in A, B, C, and D) and DAPI (blue in A’, B’, C’ and D’). Scale bar = 10 µm.

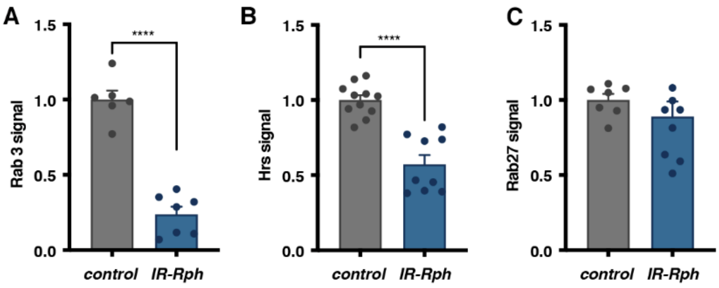

Figure S7. Interference of *Rph* decreases Rab 3 vesicles. Quantification of Rab 3 (A) Hrs (B) and Rab27 (C) signal from immunofluorescence assay of control (*Hand-Gal4>UAS-GFP yw*) and *Rph* RNAi knockdown (*Hand-Gal4>UAS-GFP UAS-IR-Rph*) pericardial nephrocytes. Student´s t-test. ****p<0.0001.

Disease Models & Mechanisms • Supplementary information

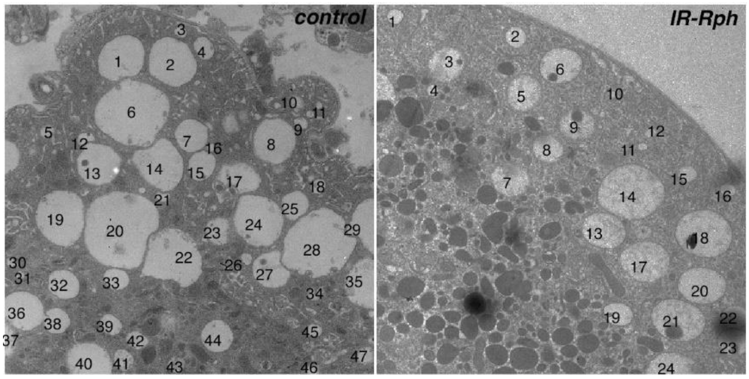

Figure S8. Downregulation of *Rph* reduces the number of endosomes in pericardial nephrocytes. Transmission electron microscopy images of control (*Hand-Gal4>UAS-GFP*) and *Rph* RNAi knockdown (*Hand-Gal4>UAS-GFP UAS-IR-Rph*) pericardial nephrocytes. Numbers indicate the numbering of endosomes.

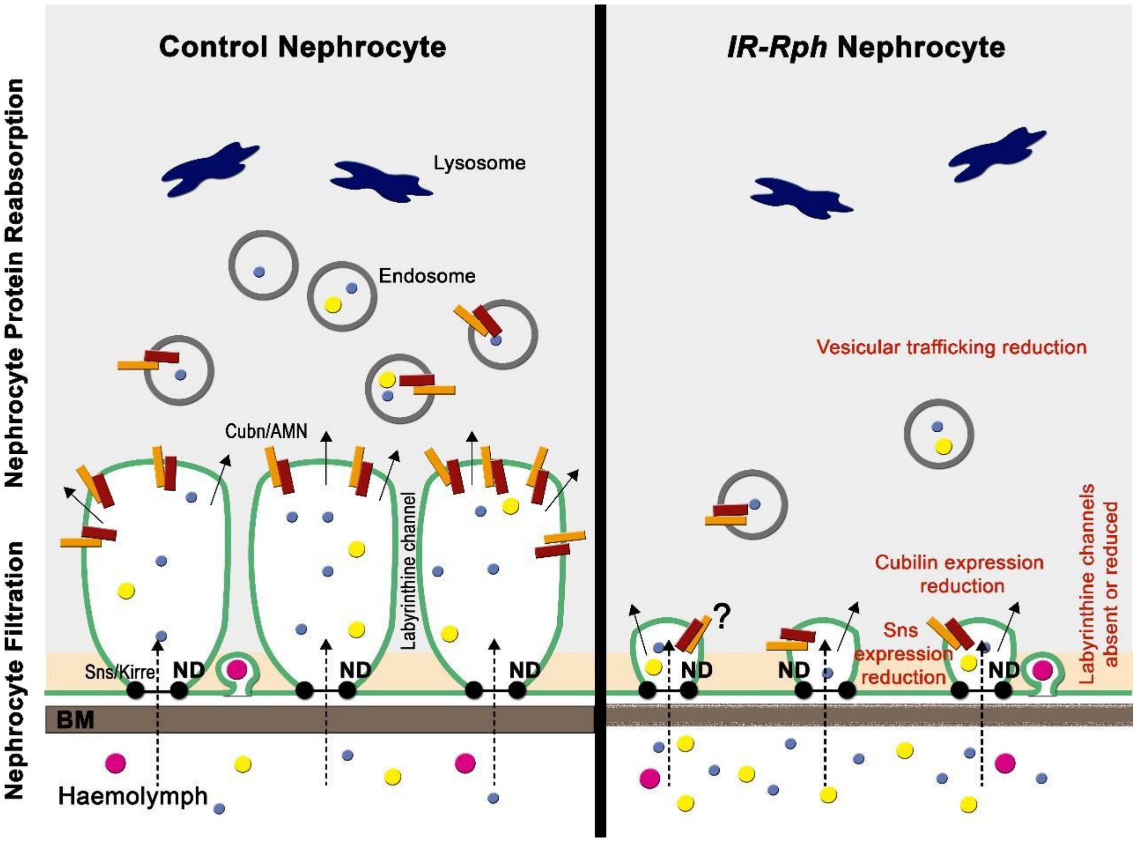

Figure S9. Model of the alterations caused by *Rph* knockdown in the nephrocyte function. The interference of *Rph* expression affects nephrocyte filtration by reducing *sns* expression, lowering nephrocyte protein reabsorption through the reduction of vesicular trafficking and *Cubilin* expression, and affecting the labyrinthine channels' structure. Nephrocyte filtration is indicated by discontinuous arrows and protein reabsorption is indicated by short arrows. The diversity of molecules present in the hemolymph is represented by colored circles of different sizes. BM, basement membrane; ND, nephrocyte diaphragm.

Disease Models & Mechanisms • Supplementary information
